# Effectiveness and Safety of Saroglitazar in Patients With Metabolic Disease in India Stratified by Estimated Glomerular Filtration Rate (eGFR): A Retrospective, Observational, Electronic Medical Record (EMR)-Based Real-World Evidence Study

**DOI:** 10.7759/cureus.105083

**Published:** 2026-03-12

**Authors:** Kamlakar Tripathi, Deodatta Chafekar, Himanshu Verma, Ganapathi Bantwal, Parag Shah, Supratik Bhattacharya, Manisha Sahay, Vishal Golay, Shanmugraj G., Rajiv Awasthi, Ashok Jaiswal, Vinodhini Mudaliyar, Kunal S Jhaveri, Jayanthy G., Snehal Shah, Garima Verma

**Affiliations:** 1 Department of Medicine, Institute of Medical Sciences, Banaras Hindu University, Varanasi, IND; 2 Department of Nephrology, Supreme Kidney Care, Nashik, IND; 3 Department of Nephrology and Renal Transplant Medicine, Vardhman Mahavir Medical College (VMMC) and Safdarjung Hospital, New Delhi, IND; 4 Department of Endocrinology, St. John's Medical College Hospital, Bengaluru, IND; 5 Department of Endocrinology, Gujarat Endocrine Centre, Ahmedabad, IND; 6 Department of Endocrinology, Diabetes and Metabolism, SKN Diabetes and Endocrine Centre, Kolkata, IND; 7 Department of Nephrology, Osmania Medical College, Hyderabad, IND; 8 Department of Nephrology, Maxwell Hospital, Siliguri, IND; 9 Department of Nephrology, Thangam Hospital, Palakkad, IND; 10 Department of Diabetes and Endocrinology, Prarthana Clinic and Diabetes Care Centre, Lucknow, IND; 11 Department of Medical Affairs, Zydus Healthcare Limited, Mumbai, IND; 12 Department of Clinical Insights, HealthPlix Technologies Private Limited, Bengaluru, IND

**Keywords:** dyslipidemia, estimated glomerular filtration rate, glycemic control, metabolic dysfunction, real world study, renal function, saroglitazar

## Abstract

Purpose

Metabolic dysfunction and chronic kidney disease (CKD) often coexist, presenting therapeutic challenges. Saroglitazar, a dual proliferator-activated receptor (PPAR)-α/γ agonist, shows promising effects in managing dyslipidemia and glycemic abnormalities; however, real-world evidence (RWE) is limited across varying degrees of renal function. This study evaluated the effectiveness and safety of saroglitazar (4 mg) in patients with metabolic disease in India stratified by estimated glomerular filtration rate (eGFR).

Methods

This retrospective observational study analyzed the electronic medical records (EMRs) of 614 patients with metabolic disorders prescribed saroglitazar. Changes in lipid and glycemic parameters, liver enzymes, and renal function were assessed from baseline to follow-up (minimum 90 days) across eGFR categories.

Results

A total of 614 patients were included. Metabolic and cardiac disorders were the most common conditions at baseline. Significant reductions were observed in triglycerides (TG, -62.68 mg/dL), low-density lipoproteins (LDL, -7.03 mg/dL), and glycosylated hemoglobin (HbA1c, -0.76%) in the overall population (p < 0.001), with similar reductions in patients with eGFR ≥90 mL/min/1.73 m² and 60-89.99 mL/min/1.73 m². Patients with hepatic conditions showed a significant decrease in aspartate transaminase (AST) and alanine transaminase (ALT) levels. eGFR levels remained stable across categories. Saroglitazar was associated with a low incidence of adverse events, including asthenia (n = 12, 1.95%) and pyrexia (n = 6, 0.97%).

Conclusion

Saroglitazar was associated with improved lipid and glycemic profiles across various levels of kidney function, with limited safety concerns. These real-world findings suggest the potential metabolic benefits of saroglitazar in Indian patients with metabolic abnormalities, across diverse renal functions.

## Introduction

Metabolic dysfunction, described as metabolic syndrome (MetS), encompasses a group of interrelated risk factors, including obesity, insulin resistance, elevated fasting glucose, dyslipidemia, and hypertension, and presents a growing public health challenge in India [1]. It is a critical subset of metabolic diseases that significantly elevates the risk of type 2 diabetes mellitus (T2DM), atherosclerotic cardiovascular disease, and chronic kidney disease (CKD) [2]. The global prevalence of MetS ranges from 12.5% to 31.4%, contingent on the diagnostic criteria used [3]. A recent meta-analysis indicated that the prevalence of MetS in India is 30%, with higher occurrence among older adults (>60 years), women, and urban populations [4].

In 2023, the American Heart Association introduced the Cardiovascular-Kidney-Metabolic (CKM) syndrome, highlighting the interconnected functions of the heart, kidneys, and metabolic system. However, this model excludes the liver, an essential organ affected by metabolic disorders [5,6]. This framework is clinically relevant, as metabolic dysfunction, metabolic dysfunction-associated steatotic liver disease (MASLD), and CKD frequently coexist and modify renal risk. Epidemiological data demonstrate an independent association between MetS and accelerated glomerular filtration rate (GFR) decline [7-10]. This context supports evaluating metabolic therapies, including saroglitazar, across estimated GFR (eGFR) strata in routine practice.

Studies have indicated that insulin resistance and hyperglycemia contribute to endothelial dysfunction and glomerular injury, while obesity and dyslipidemia promote renal damage via ultrafiltration, renin-angiotensin-aldosterone system (RAAS) activation, and lipotoxicity [11]. Given the reciprocal link between metabolic dysregulation and declining kidney function, categorizing patients by eGFR is essential for tailoring treatment plans and assessing drug tolerability. eGFR serves as a key indicator of renal health and disease progression. The National Kidney Foundation (NKF) practice guidelines classify eGFR into the following categories: ≥90 mL/min/1.73 m², 60-89.99 mL/min/1.73 m², 45-59.99 mL/min/1.73 m², 30-44.99 mL/min/1.73 m², 15-29.99 mL/min/1.73 m², and <15 mL/min/1.73 m² [12], providing a framework to assess the effectiveness of the drug and its safety across various eGFR strata.

Saroglitazar, a dual peroxisome proliferator-activated receptor (PPAR)-α/γ agonist, has been evaluated as a therapeutic option for addressing multiple metabolic derangements [13]. It has demonstrated lipid- and glycemic-lowering effects in patients with T2DM [14]. However, T2DM and dyslipidemia frequently coexist in routine clinical practice, with varying degrees of kidney function. Additionally, patients with moderate kidney function are often less represented in clinical trials. Consequently, real-world evidence (RWE) on the metabolic outcomes and renal safety of saroglitazar across different levels of kidney function remains limited. The present study aimed to evaluate the effectiveness of saroglitazar in patients with metabolic disorders, stratified by eGFR categories, as an extension of our previous LEAD INDIA electronic medical record (EMR) study [15].

## Materials and methods

Study design

This retrospective observational study analyzed anonymized EMR data to evaluate the effectiveness of saroglitazar in patients with metabolic disorders and those stratified by eGFR. Male and female patients ≥18 years of age with metabolic diseases (MASLD/T2DM/obesity/dyslipidemia) who were prescribed saroglitazar were considered for the study. Patient data were retrieved from the HealthPlix EMR database during the study period as per the inclusion criteria. The baseline visit (Visit 1) was defined as the visit where saroglitazar was initiated. Patients with recorded test values of glycosylated hemoglobin (HbA1c), aspartate transaminase (AST), alanine transaminase (ALT), low-density lipoprotein (LDL), and triglycerides (TGs) available from the baseline visit (Visit 1) and the follow-up visit (Visit 2), and eGFR at the baseline visit, were included in the study. Patients were stratified into eGFR categories at baseline according to NKF categorization. The follow-up visit (Visit 2) was defined as the first visit occurring 90 days after saroglitazar initiation with available values for HbA1c, AST, ALT, LDL, and TGs. Patients who did not meet the above criteria were excluded from the study.

The primary endpoints of this study were to evaluate changes in the levels of HbA1c, LDL, and TG from baseline to Visit 2 in patients with metabolic disorders, overall and stratified by eGFR. The secondary endpoints were the assessment of changes in the levels of high-density lipoprotein (HDL), total cholesterol (TC), non-HDL, fasting blood glucose (FBG), postprandial blood glucose (PPBG), serum creatinine, triglyceride glycemic (TyG) index, and eGFR, as well as changes in AST and ALT levels in diseased liver conditions (non-alcoholic fatty liver disease (NAFLD)/chronic liver disease/fatty liver/MASLD/steatohepatitis) from baseline to Visit 2, and the assessment of safety in metabolic patients with different eGFR strata.

Data collection and variables

Anonymized and aggregated data of patients from January 2017 to March 2025 were retrieved from the HealthPlix EMR database (https://healthplix.com/) for analysis. Baseline data included demographics (age, gender, weight, and body mass index (BMI)), lipid and glycemic parameters, liver enzyme levels, serum creatinine, and eGFR. The demographic characteristics (age, gender, weight, and BMI) were analyzed at the overall level, and the outcomes were stratified by baseline eGFR levels. Baseline comorbidities and concomitant medications were retrieved to understand the patient profile. Changes in glycemic parameters, lipid profiles, liver enzyme levels, serum creatinine, and eGFR were evaluated at the follow-up visit. The metabolic conditions (MASLD/NAFLD, T2DM, obesity, and dyslipidemia) were identified based on physician-entered diagnoses recorded in the EMR platform. Concomitant medications were identified from prescriptions documented at baseline. The complaints section of the EMR was analyzed for safety. The study was approved by the Central Independent Ethics Committee (CIEC) (IEC No: CIEC/2025/049, dated April 22, 2025).

Statistical analysis

Stata software (StataCorp LLC, College Station, TX, USA; Release 15.1 SE Limited Edition) was used for statistical analysis. Categorical variables, such as gender, were summarized as counts (n) and percentages. Continuous variables, including age, weight, TG, TC, HDL, non-HDL cholesterol, LDL, HbA1c, FBG, PPBG, AST, ALT, serum creatinine, TyG index, and eGFR, were described using descriptive statistics. These included the number of patients (n), mean, and standard deviation (SD). Changes in laboratory parameters over time were analyzed for statistical significance using the paired Student's t-test at a 5% alpha level (p ≤ 0.05). As the Bonferroni correction was overly conservative and insufficiently suited to address multiplicity arising from repeated t-tests, false discovery rate (FDR) control was applied using the Benjamini-Hochberg procedure, and results are reported as q-values. Missing values were not imputed.

## Results

Patient disposition

**Figure 1:**
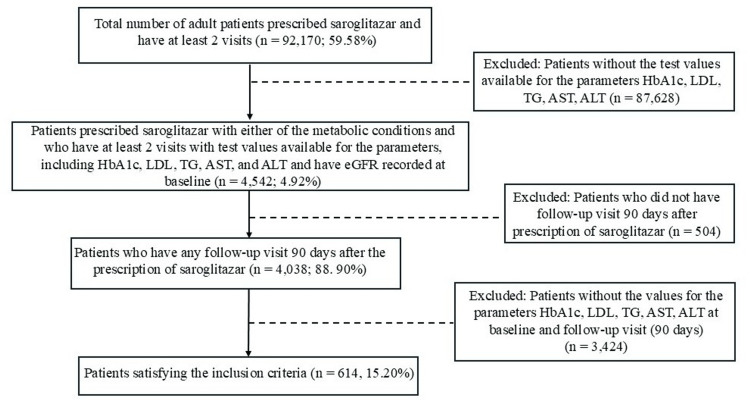
CONSORT diagram The percentage in each box was calculated using the numbers in the preceding box as the denominator. Abbreviations: n, Number of patients; HbA1c, Glycosylated hemoglobin; LDL, Low-density lipoprotein; AST, Aspartate transaminase; ALT, Alanine transaminase; eGFR, Estimated glomerular filtration rate; TG, Triglycerides

Data of 92,170 (59.58%) adult patients prescribed saroglitazar and who had at least two visits (January 2017 to March 2025) recorded in the EMR were analyzed. Among them, the laboratory values for HbA1c, AST, ALT, LDL, and TGs at two visits, and eGFR at the baseline visit, were available for 4,542 (4.92%) patients. In this group, 4,038 (88.90%) patients had at least one follow-up visit 90 days after the prescription of saroglitazar. Furthermore, 614 (15.20%) patients met the eligibility criteria, with values for HbA1c, LDL, TG, AST, and ALT recorded at baseline and at the 90-day follow-up visit. Patient disposition is illustrated in the CONSORT diagram (Figure 1). 

Baseline characteristics

Data of patients prescribed saroglitazar (n = 614) with available baseline and follow-up values for HbA1c, LDL, TGs, AST, and ALT, and eGFR (baseline only) were analyzed. The median duration of saroglitazar treatment was 295 days. The mean age of these patients was 50.61 years (SD 11.02), with 48.05% (n = 295) falling within the 18-49 years age category. Males constituted 63.68% (n = 391) of the total patient group. The mean body weight (n = 508) was 77.59 (12.77) kg. Among the 411 patients with recorded BMI, the mean was 29.33 (4.71) kg/m², with 83.94% classified as obese (BMI ≥ 25.00 kg/m²). The mean eGFR (n = 614) was 94.47 ± 21.47 mL/min/1.73 m², suggesting that kidney function was largely maintained in the study population. Most patients (63.68%, n = 391) had eGFR values ≥ 90 mL/min/1.73 m², with a mean of 107.78 ± 10.58 mL/min/1.73 m², indicating normal renal function. Patients with eGFR 60-89.99 mL/min/1.73 m² (n = 174) had a mean of 77.37 ± 8.60 mL/min/1.73 m², indicating a mild reduction in kidney function. Due to a lack of adequate sample size, patients (n = 3) with eGFR < 30 mL/min/1.73 m² were excluded from further statistical analysis. Cardiac disorders and metabolic conditions were the most prevalent, occurring in 45.76% and 42.32% of patients, respectively. The median duration of saroglitazar treatment (n = 614) was 295 days, with most patients receiving it for 169-571 days (Table 1).

**Table 1:** Baseline characteristics Percentages were calculated by using 614 as the denominator. ^1^Diabetes mellitus, dyslipidemia, non-alcoholic fatty liver disease (NAFLD), chronic liver disease, fatty liver/metabolic dysfunction-associated steatotic liver disease (MASLD), steatohepatitis; ^2^Hypertension, ischemic heart disease, coronary artery disease; ^3^Microalbuminuria, nephropathy Abbreviations: n: Number of patients; BMI: Body mass index; eGFR: Estimated glomerular filtration rate

Demographic characteristics				
Variable		n	%	Mean ± SD
Age (years)	Overall	614	100	50.61 ± 11.02
	18 - 49	295	48.05	41.21 ± 5.47
	50 - 59	179	29.15	54.31 ±2.92
	≥60	140	22.80	65.67 ± 5.33
Weight (kg)	Overall	508	100	77.59 ± 12.77
BMI (kg/m^2^)	Overall	411	100	29.33 ± 4.71
	Underweight (<18.50)	3	0.73	17.27 ± 1.14
	Normal (18.50 to 22.99)	22	5.35	21.72 ± 1.19
	Overweight (23 to 24.99)	41	9.98	24.03 ± 0.67
	Obese (≥25)	345	83.94	30.55 ± 4.07
Gender	Overall	614	100	-
	Male	391	63.68	
	Female	223	36.32	
Clinical characteristics				
eGFR (mL/min/1.73 m^2^)	Overall	614	100	94.47 ± 21.47
	≥90	391	63.68	107.78 ± 10.58
	60-89.99	174	28.34	77.37 ± 8.60
	45-59.99	37	6.03	53.29 ± 4.85
	30-44.99	9	1.47	39.99 ± 4.54
	15-29.99	3	0.49	22.65 ± 5.98
Conditions at baseline				
Metabolic conditions^1^		260	42.32	-
Cardiac disorders^2^		281	45.76	
Kidney diseases^3^		24	3.91	
Others		121	19.7	

Concomitant medications

The data were analyzed to evaluate co-prescribed medications. Antidiabetic medications were the most commonly recommended (n = 1,120), followed by lipid-lowering drugs (n = 388) and antihypertensive medications (n = 281). Among the antidiabetic agents, a combination of sulfonylureas and biguanides (17.40%) was the most commonly recommended (Table 2).

**Table 2:** Concomitant medications at baseline Others include the medications that were less frequently prescribed. Percentages were calculated by using the therapy patient count as the denominator Abbreviations: n: Number of patients; DPP4: Dipeptidyl peptidase 4; SGLT2: Sodium-glucose co-transporter 2; ARB: Angiotensin receptor blockers; CCB: Calcium channel inhibitors; GLP1: Glucagon-like peptide 1

Drug class	Therapy type	Medications	n	%
Antidiabetic agents (n = 1120)	Single agent	DPP4 inhibitors	122	10.89
		Sulfonylurea	73	6.52
		Biguanides	61	5.45
		Insulin glargine	43	3.84
		SGLT2 inhibitors	72	6.42
		Insulin	35	3.13
		Alpha-glucosidase inhibitors	24	2.14
		Thiazolidinediones	16	1.42
		GLP1 receptor agonist	17	1.25
	Dual agents	Sulfonylurea + biguanides	196	17.50
		DPP4 inhibitors + biguanides	171	15.26
		SGLT2 + DPP4 inhibitors	92	8.21
		SGLT2 + biguanides	41	3.66
		Insulin aspart + insulin aspart protamine	10	0.89
		Insulin aspart + Insulin degludec	18	1.60
		Human insulin + Nph human insulin	16	1.42
		Others	14	1.25
	Triple agents	Sulfonylurea + biguanide + alpha-glucosidase inhibitor	44	3.93
		SGLT2 inhibitors + biguanide + DPP4 inhibitors	30	2.62
		Sulfonylurea + biguanide + thiazolidinedione	14	1.25
		Others	11	1.00
Antihyperlipidemic agents (n = 388)	Single agent	Statins	246	63.4
	Dual agents	Antiplatelets + statins	90	23.19
		Fibrates + statins	43	11.09
		Cholesterol absorption inhibitor + statins	9	2.32
Antihypertensive (n = 281)	Single agent	ARB	82	29.18
		Beta-blockers	36	12.81
		CCB	32	11.38
	Dual agents	CCB + ARB	51	18.14
		Beta-blockers + ARB	18	6.41
		Chlorthalidone + ARB	17	6.05
		CCB + beta-blockers	14	4.09
		Others	31	1.03

Primary endpoints

The primary endpoints were changes in TG, LDL, and HbA1c (Table 3).

**Table 3:** Change in the TG, LDL, and HbA1c levels *p-values, except for the overall comparison, were evaluated using a Bonferroni-adjusted significance level to control for inflation of Type I error (α). The Bonferroni-adjusted significance threshold was α = 0.01. For the p-value related to the overall section significance threshold was α = 0.05. The q-value was calculated using False Discovery Rate (FDR) control with the Benjamini-Hochberg (BH) procedure. q-values were not calculated for the overall section of the table. They were only calculated for sections where the p-value threshold was recalculated using the Bonferroni-adjusted method. ^†^q-values were significant at the 0.05 level of significance. The subgroup with a smaller number of patients was interpreted cautiously. Abbreviations: n: Number of patients; TG: Triglycerides; SD: Standard deviation; eGFR: Estimated glomerular filtration rate; NS: Non-significant; HbA1c: Glycated hemoglobin; LDL: Low-density lipoprotein

Parameter	Category of eGFR (mL/min/1.73 m^2^)	n	Baseline, mean ± SD	Follow-up, mean ± SD	Change from baseline		t-statistic value	p-value	q-value
					Mean	%			
TG (mg/dL)	Overall	614	217.71 ± 102.03	155.03 ± 80.16	-62.68	-28.79	-16.15	<0.001^*^	-
	≥90	391	221.45 ± 103.58	156.47 ± 83.22	-64.98	-29.34	-12.98	<0.001^*^	0.0020^†^
	60-89.99	174	208.02 ± 94.83	151.24 ± 77.20	-56.78	-27.30	-8.60	<0.001^*^	0.0020^†^
	45-59.99	37	217.79 ± 112.80	161.68 ± 66.31	-56.11	-25.76	-3.38	0.0017^*^	0.0023^†^
	30-44.99	9	208.31 ± 114.95	122.03 ± 55.13	-86.28	-41.42	-2.19	0.0596 (NS)	0.0596 (NS)
LDL (mg/dL)	Overall	614	92.21 ± 36.50	85.18 ± 31.15	-7.03	-7.62	-4.68	<0.001^*^	-
	≥90	391	94.76 ± 36.70	87.70 ± 31.66	-7.06	-7.45	-3.72	<0.001^*^	0.0040^†^
	60-89.99	174	88.89 ± 37.54	81.30 ± 30.44	-7.59	-8.53	-2.76	0.0065^*^	0.0130^†^
	45-59.99	37	84.60 ± 28.53	80.32 ± 28.15	-4.28	-5.05	-0.66	0.5154 (NS)	0.5154 (NS)
	30-44.99	9	84.16 ± 31.45	69.28 ± 19.52	-14.88	-17.68	-1.32	0.2220 (NS)	0.0795 (NS)
HbA1c (%)	Overall	614	8.18 ± 1.82	7.42 ± 1.50	-0.76	-9.29	-10.99	<0.001^*^	-
	≥90	391	8.18 ± 1.84	7.36 ± 1.52	-0.82	-10.02	-9.41	<0.001^*^	0.0020^†^
	60-89.99	174	8.10 ± 1.80	7.45 ± 1.52	-0.65	-8.02	-4.93	<0.001^*^	0.0020^†^
	45-59.99	37	8.36 ± 1.67	7.53 ± 1.16	-0.83	-9.93	-2.68	0.0111 (NS)	0.0148^†^
	30-44.99	9	8.57 ± 1.84	7.99 ± 1.60	-0.58	-6.77	-0.99	0.3473 (NS)	0.3473 (NS)

At follow-up (any visit within 90 days after prescription of saroglitazar), statistically significant reductions in TG levels were observed in the overall population (p < 0.001; n = 614). This was evident across the eGFR categories ≥ 90 and 60-89.99 mL/min/1.73 m², with statistically significant reductions (p < 0.001) observed after Bonferroni correction. Although the decrease in the lower eGFR category of 30-44.99 mL/min/1.73 m² was notable, it did not reach statistical significance after correction.

LDL levels decreased significantly in the overall population (p < 0.001; n = 614) from baseline to follow-up, with significant reductions in patients with eGFR ≥ 90 mL/min/1.73 m² (n = 391) and eGFR 60-89.99 mL/min/1.73 m² (n = 174). Reductions in the lower eGFR categories did not reach statistical significance after adjustment.

In the overall population (n = 614), a decrease in the mean HbA1c level was observed from 8.18% to 7.42% (-0.76%, p < 0.001). A similar trend was observed in the eGFR subgroups of ≥ 90 mL/min/1.73 m² (n = 391) and 60-89.99 mL/min/1.73 m² (n = 174).

Secondary endpoints

Change in HDL, TC, and Non-HDL

A modest improvement in HDL (1.43 mg/dL) was seen in patients with eGFR 60-89.99 mL/min/1.73 m² (n = 160), though this was not statistically significant. Those with eGFR ≥ 90 mL/min/1.73 m² (n = 359) exhibited no significant change. TC demonstrated a significant mean reduction in the overall population (n = 46; p = 0.0249), with consistent but nonsignificant reductions across all eGFR categories. The non-HDL levels exhibited a modest, nonsignificant overall decline (-5.65 mg/dL; n = 41). The mean changes in HDL, TC, and non-HDL are illustrated in Table 4.

**Table 4:** Change in HDL, TC, and non-HDL *p-values, except for the overall comparison, were evaluated using a Bonferroni-adjusted significance level to control for inflation of Type I error (α). The Bonferroni-adjusted significance threshold was α = 0.01. For the p-value related to the overall section, the significance threshold was α = 0.05. The q-value was calculated using False Discovery Rate (FDR) control with the Benjamini-Hochberg (BH) procedure. q-values were not calculated for the overall section of the table. They were only calculated for sections where the p-value threshold was recalculated using the Bonferroni-adjusted method. Abbreviations: n: Number of patients; HDL: High-density lipoprotein; TC: Total cholesterol; SD: Standard deviation; NS: Non-significant

Parameter	Category of eGFR (mL/min/1.73m^2^)	n	Baseline, mean ± SD	Follow-up, mean ± SD	Change from the baseline		t-statistic value	p-value	q-value
					Mean	%			
HDL (mg/dL)	Overall	565	41.61 ± 08.61	42.04 ± 9.69	0.43	1.03	-1.14	0.2542 (NS)	-
	≥90	359	41.61 ± 8.44	41.93 ± 9.37	0.32	0.77	-0.70	0.4836 (NS)	0.4836 (NS)
	60-89.99	160	41.39 ± 8.93	42.82 ± 10.41	1.43	3.45	-1.98	0.0499 (NS)	0.1996 (NS)
	45-59.99	36	43.24 ± 7.18	40.98 ± 10.01	-2.26	-5.23	1.61	0.1171 (NS)	0.2342 (NS)
	30-44.99	9	41.94 ± 11.88	38.26 ± 6.89	-3.68	-8.77	0.97	0.3611 (NS)	0.4815 (NS)
TC (mg/dL)	Overall	46	172.24 ± 43.92	160 ± 42.91	-12.24	-7.11	-2.32	0.0249^*^	-
	≥90	23	177.54 ± 48.46	163.87 ± 43.07	-13.67	-7.70	-1.66	0.1103 (NS)	0.3102 (NS)
	60-89.99	14	164.78 ± 41.74	152.19 ± 41.88	-12.59	-7.64	-1.33	0.2068 (NS)	0.3102 (NS)
	45-59.99	9	170.30 ± 37.18	162.26 ± 47.41	-8.04	-4.72	-0.82	0.4361 (NS)	0.4361 (NS)
Non-HDL (mg/dL)	Overall	41	126.03 ± 36.02	120.38 ± 40.50	-5.65	-1.11	0.2739 (NS)	0.2739 (NS)	-
	≥90	18	128.54 ± 39.25	123.94 ± 42.00	-4.60	-0.55	0.5893 (NS)	0.5893 (NS)	0.5893 (NS)
	60-89.99	14	121.30 ± 36.59	113.85 ± 41.38	-7.45	-0.77	0.4540 (NS)	0.4540 (NS)	0.5893 (NS)
	45-59.99	9	128.36 ± 31.26	123.42 ± 39.46	-4.94	-0.66	0.5304 (NS)	0.5304 (NS)	0.5893 (NS)

Change in TG in Patients With Baseline LDL <100 mg/dL and TG >150 mg/dL

A significant reduction of -92.26 mg/dL was observed in this subset of 244 patients with baseline LDL < 100 mg/dL and TG > 150 mg/dL (p < 0.001). Significant reductions were also observed across most eGFR strata, including patients with eGFR ≥ 90 mL/min/1.73 m² (n = 150), 60-89.99 mL/min/1.73 m² (n = 71), and 45-59.99 mL/min/1.73 m² (n = 16) (all p < 0.001 after Bonferroni adjustment). The reduction in the eGFR 30-44.99 mL/min/1.73 m² subgroup (n = 4) did not remain statistically significant after adjustment, likely due to the small sample size (Table 5).

**Table 5:** TG levels in patients with baseline LDL < 100 mg/dL and TG > 150 mg/dL *p-values, except for the overall comparison, were evaluated using a Bonferroni-adjusted significance level to control for inflation of Type I error (α). The Bonferroni-adjusted significance threshold was α = 0.01. For the p-value related to the overall section, the significance threshold was α = 0.05. The q-value was calculated using False Discovery Rate (FDR) control with the Benjamini-Hochberg (BH) procedure. q-values were not calculated for the overall section of the table. They were only calculated for sections where the p-value threshold was recalculated using the Bonferroni-adjusted method. ^†^q-values were significant at the 0.05 level of significance. The subgroup with a smaller number of patients was interpreted cautiously. Abbreviations: n: Number of patients; TG: Triglycerides; SD: Standard deviation; eGFR: Estimated glomerular filtration rate; NS: Non-significant

Parameter	Category of eGFR (mL/min/1.73 m^2^)	n	Baseline, mean ± SD	Follow-up, mean ± SD	Change from the baseline		t-statistic value	p-value	q-value
					Mean	%			
TG (mg/dL)	Overall	244	269.37 ± 87.05	177.11 ± 82.77	-92.26	-34.25	-15.49	<0.001^*^	-
	≥90	150	275.21 ± 92.23	179.10 ± 86.88	-96.11	-34.92	-12.01	<0.001^*^	<0.001^†^
	60-89.99	71	254.58 ± 74.65	173.81 ± 82.34	-80.77	-31.72	-7.63	<0.001^*^	<0.001^†^
	45-59.99	16	285.53 ± 86.84	176.95 ± 47.82	-108.58	-38.02	-6.03	<0.001^*^	<0.001^†^
	30-44.99	4	233.25 ± 55.17	141.25 ± 82.07	-92	-39.44	-5.73	0.0106 (NS)	0.0159^†^

Change in FBG, PPBG Levels, and TyG Index

FBG levels showed a significant decrease across all eGFR categories, with greater reductions observed in the ≥ 90 and 60-89.99 mL/min/1.73 m² groups (-29.12 mg/dL and -19.90 mg/dL). The reductions in the other eGFR categories were smaller and did not reach statistical significance, which might be due to the smaller sample size. The PPBG levels declined in the overall population and in the eGFR ≥ 90, 60-89.99, and 45-59.99 mL/min/1.73 m² subgroups, whereas an increase was observed in the 30-44.99 mL/min/1.73 m² subgroup, which should be interpreted with caution due to the small sample size. The TyG index showed a decline in the overall population and across the eGFR subgroups of ≥ 90 and 60-89.99 mL/min/1.73 m². The changes in the FBG, PPBG levels, and TyG index are represented in Table 6.

**Table 6:** Change in FBG, PPBG levels, and TyG index *p-values, except for the overall comparison, were evaluated using a Bonferroni-adjusted significance level to control for inflation of Type I error (α). The Bonferroni-adjusted significance threshold was α = 0.01. For the p-value related to the overall section, the significance threshold was α = 0.05. The q-value was calculated using False Discovery Rate (FDR) control with the Benjamini-Hochberg (BH) procedure. q-values were not calculated for the overall section of the table. They were only calculated for sections where the p-value threshold was recalculated using the Bonferroni-adjusted method. ^†^q-values were significant at the 0.05 level of significance. The subgroup with a smaller number of patients was interpreted cautiously. Abbreviations: n: Number of patients; SD: Standard deviation; PPBG: Post-prandial blood glucose; FBG: Fasting blood glucose; TyG: Triglyceride-glucose index; eGFR: Estimated glomerular filtration rate

Parameters	Category of eGFR (mL/min/1.73 m^2^)	n	Baseline, mean ± SD	Follow-up, mean ± SD	Change from baseline		t-statistic value	p-value	q-value
					Mean	%			
FBG (mg/dL)	Overall	482	158.21 ± 57.09	132.45 ± 44.36	-25.76	-16.28	-9.51	<0.001^*^	-
	≥90	312	162.08 ± 58.27	132.96 ± 42.19	-29.12	-17.97	-8.91	<0.001^*^	0.0020^†^
	60-89.99	133	151.29 ± 53.70	131.39 ± 51.57	-19.90	-13.15	-3.53	<0.001^*^	0.0020^†^
	45-59.99	28	139.99 ± 41.73	128.68 ± 32.22	-11.31	-8.08	-1.16	0.2564 (NS)	0.2564 (NS)
	30-44.99	8	158.70 ± 45.55	130.48 ± 19.54	-28.22	-17.78	-2.34	0.0518 (NS)	0.0691 (NS)
PPBG (mg/dL)	Overall	194	218.62 ± 77.28	182.04 ± 61.29	-36.58	-16.73	-5.96	<0.001^*^	-
	≥90	118	218.41 ± 72.78	178.87 ± 51.38	-39.54	-18.10	-5.58	<0.001^*^	0.0040^†^
	60-89.99	60	222.82 ± 89.47	186.91 ± 79.36	-35.91	-16.12	-2.70	0.0090^*^	0.0180^†^
	45-59.99	14	203.44 ± 55.70	178.43 ± 50.39	-25.01	-12.29	-1.34	0.2021 (NS)	0.2695 (NS)
	30-44.99	2	211 ± 125.87	248.50 ± 2.12	37.50	-17.77	0.43	0.7422 (NS)	0.7422 (NS)
TyG Index	Overall	482	9.56 ± 0.62	9.06 ± 0.61	-0.50	-5.23	-16.61	<0.001^*^	-
	≥90	312	9.60 ± 0.63	9.07 ± 0.61	-0.53	-5.52	-14.48	<0.001^*^	0.0020^†^
	60-89.99	133	9.48 ± 0.60	9.02 ± 0.64	-0.46	-4.85	-7.73	<0.001^*^	0.0020^†^
	45-59.99	28	9.37 ± 0.51	9.16 ± 0.50	-0.21	-2.24	-2.19	0.0368 (NS)	0.0467^†^
	30-44.99	8	9.51 ± 0.73	8.86 ± 0.40	-0.65	-6.83	-2.41	0.0467 (NS)	0.0467^†^

Change in the AST and ALT in Patients With Diseased Liver Conditions

In the overall population with diseased liver conditions (n = 197), including NAFLD, chronic liver disease, fatty liver, MASLD, or steatohepatitis as recorded in the EMR, significant reductions in AST and ALT levels were observed from baseline (p < 0.001). Similar decreases were noted in patients with eGFR ≥ 90 mL/min/1.73 m² and 60-89.99 mL/min/1.73 m² (p < 0.001). For those with eGFR 45-59.99 mL/min/1.73 m², numerical reductions in AST and ALT were observed but did not reach statistical significance (Table 7).

**Table 7:** Change in the AST and ALT in patients with diseased liver conditions *p-values, except for the overall comparison, were evaluated using a Bonferroni-adjusted significance level to control for inflation of Type I error (α). The Bonferroni-adjusted significance threshold was α = 0.01. For the p-value related to the overall section, the significance threshold was α = 0.05. The q-value was calculated using False Discovery Rate (FDR) control with the Benjamini-Hochberg (BH) procedure. q-values were not calculated for the overall section of the table. They were only calculated for sections where the p-value threshold was recalculated using the Bonferroni-adjusted method. ^†^q-values were significant at the 0.05 level of significance. The subgroup with a smaller number of patients was interpreted cautiously. Abbreviations: n: Number of patients; AST: Aspartate transaminase; ALT: Alanine aminotransferase; eGFR: Estimated glomerular filtration rate; SD: Standard deviation; NS: Non-significant

Parameters	Category of eGFR (mL/min/1.73 m^2^)	n	Baseline, mean ± SD	Follow-up, mean ± SD	Change from baseline		t-statistic value	p-value	q-value
					Mean	%			
AST (IU/L)	Overall	197	36.23 ± 11.01	29.38 ± 9.17	-6.85	-18.91	-8.59	<0.001^*^	-
	≥90	136	35.59 ± 10.87	28.86 ± 8.89	-6.73	-18.91	-6.99	<0.001^*^	0.0015^†^
	60-89.99	50	38.69 ± 10.91	30.80 ± 10.29	-7.89	-20.39	-4.69	<0.001^*^	0.0015^†^
	45-59.99	10	32.75 ± 12.96	29.63 ± 07.06	-3.12	-9.53	-1.39	0.7774 (NS)	0.7774 (NS)
ALT (IU/L)	Overall	197	48.13 ± 18.49	32.22 ± 14.41	-15.91	-33.06	-12.11	<0.001^*^	-
	≥90	136	48.82 ± 18.40	32.67 ± 14.57	-16.15	-33.08	-10.04	<0.001^*^	0.0015^†^
	60-89.99	50	47.92 ± 18.13	32.23 ± 14.72	-15.69	-32.74	-6.16	<0.001^*^	0.0015^†^
	45-59.99	10	42.39 ± 21.41	27.27 ± 10.49	-15.12	-35.67	-2.59	0.0289 (NS)	0.0289^†^

Changes in eGFR and Serum Creatinine

**Figure 2:**
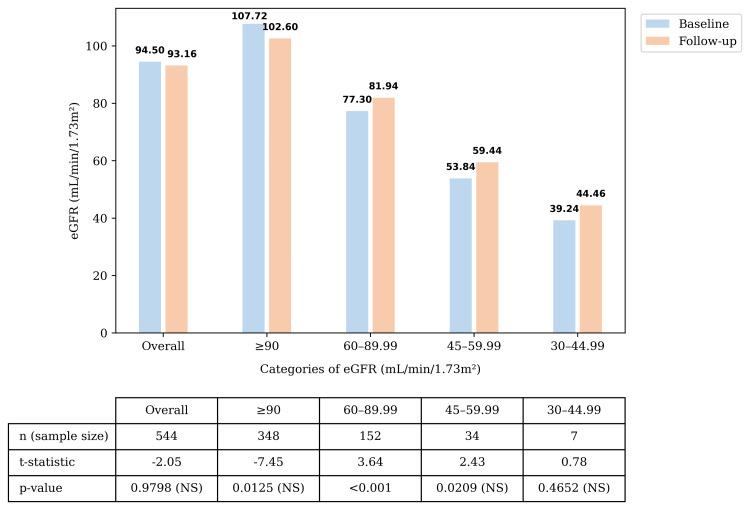
Change in the eGFR Change in eGFR levels across the eGFR categories from baseline to follow-up. p-values, except for the overall comparison, were evaluated using a Bonferroni-adjusted significance level to control for inflation of Type I error (α). The Bonferroni-adjusted significance threshold was α = 0.01. For the p-value related to the overall section, the significance threshold was α = 0.05. The subgroup with a smaller number of patients was interpreted cautiously. Abbreviations: eGFR: Estimated glomerular filtration rate; n: Number of patients; NS: Non-significant

**Figure 3:**
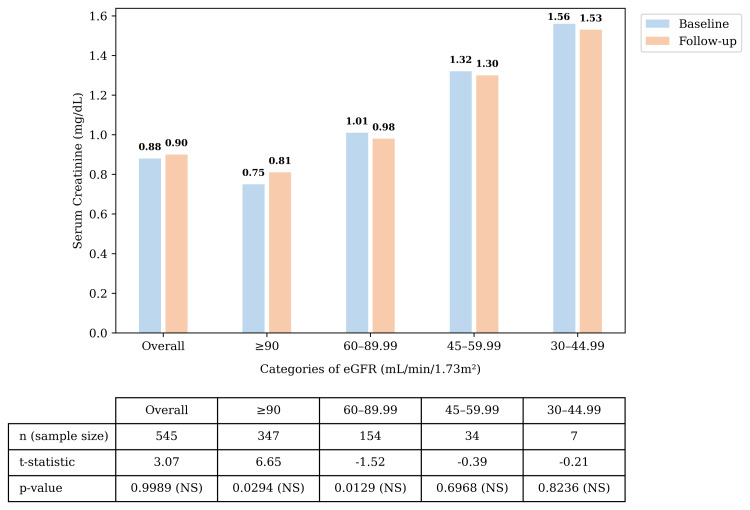
Change in serum creatinine Change in serum creatinine across the eGFR categories from baseline to follow-up. p-values, except for the overall comparison, were evaluated using a Bonferroni-adjusted significance level to control for inflation of Type I error (α). The Bonferroni-adjusted significance threshold was α = 0.01. For the p-value related to the overall section, the significance threshold was α = 0.05. The subgroup with a smaller number of patients was interpreted cautiously. Abbreviations: eGFR: Estimated glomerular filtration rate; n: Number of patients; NS: Non-significant

The overall population (n = 544) exhibited minimal, non-significant change (-1.34 mL/min/1.73 m²) in the mean eGFR from baseline to follow-up. Patients in the lower eGFR categories (45-59.99 and 30-44.99 mL/min/1.73 m²) showed modest, nonsignificant changes. In the eGFR subgroup of 60-89.99 mL/min/1.73 m², the observed increase reached statistical significance; however, this does not establish causal treatment-related renal benefits. The non-significant changes in the other eGFR categories might be due to the smaller subgroup sizes. The serum creatinine levels in the overall population and in the groups stratified by eGFR showed a non-significant mean change (Figures 2-3). 

Transition in eGFR Categories

Table 8 illustrates the changes in eGFR categories between baseline and follow-up. Across the eGFR categories, the majority of patients either remained stable or showed altered renal function at follow-up. Among those with baseline 60-89.99 mL/min/1.73 m² (n = 152), the majority of patients remained stable (63.8%), and 29.6% showed improvement. Similarly, improvement was seen in nearly half of the patients (47.1%) with baseline eGFR 45-59.99 mL/min/1.73 m² (n = 34), where 41.2% showed stable eGFR, and 11.8% declined. In the eGFR 30-44.99 mL/min/1.73 m² subgroup (n = 7), most patients remained stable (71.4%).

**Table 8:** Transition of eGFR categories Note: Stable refers to patients who remained in the same eGFR category at baseline and follow-up; Improved refers to patients whose eGFR moved to a higher category at follow-up compared to baseline; Declined refers to patients whose eGFR moved to a lower category at follow-up compared to baseline. Abbreviations: eGFR: Estimated glomerular filtration rate; n: Number of patients

Baseline eGFR (mL/min/1.73 m²)	Follow-up						
	Stable		Improved		Declined		Total
	n	%	n	%	n	%	
60-89.99	97	63.8	45	29.6	10	6.6	152
45-59.99	14	41.2	16	47.1	4	11.8	34
30-44.99	5	71.4	1	14.3	1	14.3	7

Safety Measurement of Saroglitazar

Among the 614 patients, the most commonly reported adverse events were asthenia (n = 12, 1.95%), pyrexia (n = 6, 0.97%), and gastritis (n = 5, 0.81%). Isolated cases of myalgia and vomiting (each 0.16%) were observed, while no cases of myopathy or rash were reported (Table 9).

**Table 9:** Tolerability of saroglitazar Percentages were calculated by using 614 as the denominator. Abbreviation: n: Number of patients

Event	n	%
Asthenia	12	1.95
Pyrexia	6	0.97
Gastritis	5	0.81
Myalgia	1	0.16
Vomiting	1	0.16

## Discussion

Kidney dysfunction is often accompanied by metabolic issues, such as insulin resistance and elevated blood sugar levels, indicating a complex, bidirectional relationship between CKD and the metabolic component of CKM syndrome [16]. While CKD may exacerbate metabolic problems, CKM-related metabolic dysfunction is recognized as a risk factor for both the onset and advancement of kidney disease [17]. Research has also shown that components of CKM, such as prediabetes, hypertension, and obesity, are linked to renal dysfunction [18-20]. This interplay emphasizes the importance of therapeutic approaches that can target multiple metabolic pathways while maintaining renal function. This EMR-based, real-world study provides insights into observed metabolic changes and renal function measures associated with saroglitazar prescription in Indian patients with metabolic dysfunction across different eGFR categories.

Saroglitazar, a dual PPARα/γ agonist, has been shown to effectively improve lipid and glycemic parameters [21]. Jain et al. (2019) conducted a human euglycemic clamp study involving saroglitazar in patients with T2DM and high TG levels. After four months, saroglitazar led to a significant reduction in TG and HbA1c levels compared to baseline [22]. A similar result was obtained in an Indian observational study, where a significant reduction in HbA1c and TG levels upon saroglitazar therapy was seen, without any effect on kidney function or other adverse events during six months of follow-up [23]. Consistent with this, the present study observed reductions in lipid and glycemic parameters across eGFR categories. Notably, the lipid-lowering effects were attributed to the reduction in TG levels, while changes in LDL were modest, and HDL changes were small and non-significant. Additionally, a significant reduction in TGs was observed in patients with baseline LDL < 100 mg/dL and TG > 150 mg/dL, which underscores the role of saroglitazar in managing dyslipidemia commonly associated with CKM and kidney dysfunction.

The overall HbA1c reduction was -0.76%, indicating improvement in glycemic control. Reductions in FBG and PPBG, and TyG index, highlight the role of saroglitazar in enhancing insulin sensitivity and overall metabolic regulation. These findings align with the results of the PRESS V and STOP-D studies, which illustrated the potential of saroglitazar in improving glycemic control [24,25]. Collectively, these findings suggest the dual benefits of saroglitazar on lipid and glucose metabolism.

While elevated transaminase levels do not directly indicate liver fibrosis, they are commonly used as surrogate markers for liver cell damage in MASLD, due to their accessibility and low cost [26]. The observed reductions in liver enzymes - AST and ALT - in diseased liver conditions across the eGFR categories in this study suggest associated hepatoprotective benefits, which could be particularly important given the high prevalence of MASLD in patients with CKM syndrome. These findings are concurrent with those of Goyal et al. (2020) and Bandyopadhyay et al. (2023), who noted prominent changes in hepatic enzymes [27,28].

According to the Kidney Disease Improving Global Outcomes (KDIGO), CKD is defined as abnormalities of kidney structure or function, present for ≥3 months, with health implications, commonly based on GFR and albuminuria levels [29]. In the present study, serum creatinine and eGFR remained stable across most categories. The findings indicate that saroglitazar was not associated with major observed changes in renal function measures, even in patients with lower eGFR (>30 mL/min/1.73 m²). A similar stable outcome was reported by Rodriguez-Gutierrez et al. (2022) [21]. Notably, lipid-lowering drugs are also known to improve proteinuria and, to some extent, eGFR, through reductions in inflammatory markers, though these markers were not evaluated in the present study [30]. It is recognized, however, that serum creatinine may remain stable even with worsening kidney disease. Nevertheless, the findings are reassuring in routine clinical practice, particularly in the Indian context, where diabetes, dyslipidemia, and renal impairment overlap [31].

Additionally, the low incidence of adverse events did not reveal any unexpected safety concerns. This is similar to a clinical trial involving saroglitazar over a 12-week period, which reported mild-to-moderate pyrexia, dyspepsia, and gastritis [32]. Many patients were observed to be on antidiabetic, antihyperlipidemic, and antihyperglycemic medications in conjunction with saroglitazar, which may have influenced the observed outcomes, consistent with the findings of Goyal et al. (2020), who noted a similar trend in the use of these concurrent medications [27].

Despite these promising findings, this study has certain limitations. The retrospective nature of the study and the lack of a control group limit causal inferences regarding the effectiveness of saroglitazar. The small sample sizes in the lower eGFR categories (45-59.99 and 30-44.99 mL/min/1.73 m²) limit conclusions about effectiveness. Furthermore, the NKF guidelines were followed for eGFR stratification instead of the KDIGO guidelines, due to the non-availability of data on albumin levels. In addition, multivariate analysis or stratified results by disease severity were not performed, and the interpretation of outcomes is limited due to the absence of a control or comparator arm. Since this is an EMR study, there is potential for missing data and variability in laboratory methods or recording standards. Drug compliance could not be assessed, as the study involves analysis of previously documented data. Furthermore, potential confounding by concomitant medications and comorbid conditions might have influenced the observed outcomes. 

## Conclusions

In conclusion, this real-world, EMR-based study describes observed changes in lipid and glycemic parameters and renal function measures in patients prescribed saroglitazar. Reductions in lipid and glycemic parameters, along with stable renal function, were observed, as well as changes in liver enzymes in patients with hepatic conditions. These findings suggest potential metabolic improvements associated with saroglitazar in addressing the multifaceted metabolic challenges among the Indian population.
